# Understanding the role of mechanics in nucleocytoplasmic transport

**DOI:** 10.1063/5.0076034

**Published:** 2022-06-29

**Authors:** Ion Andreu, Ignasi Granero-Moya, Sergi Garcia-Manyes, Pere Roca-Cusachs

**Affiliations:** 1 Institute for Bioengineering of Catalonia (IBEC), The Barcelona Institute of Technology (BIST), 08028 Barcelona, Spain; 2 Universitat de Barcelona, 08036 Barcelona, Spain; 3 Department of Physics, Randall Centre for Cell and Molecular Biophysics, King's College London, WC2R 2LS London, United Kingdom; Single Molecule Mechanobiology Laboratory, The Francis Crick Institute, NW1 1AT London, United Kingdom

## Abstract

Cell nuclei are submitted to mechanical forces, which in turn affect nuclear and cell functions. Recent evidence shows that a crucial mechanically regulated nuclear function is nucleocytoplasmic transport, mediated by nuclear pore complexes (NPCs). Mechanical regulation occurs at two levels: first, by force application to the nucleus, which increases NPC permeability likely through NPC stretch. Second, by the mechanical properties of the transported proteins themselves, as mechanically labile proteins translocate through NPCs faster than mechanically stiff ones. In this perspective, we discuss this evidence and the associated mechanisms by which mechanics can regulate the nucleo-cytoplasmic partitioning of proteins. Finally, we analyze how mechanical regulation of nucleocytoplasmic transport can provide a systematic approach to the study of mechanobiology and open new avenues both in fundamental and applied research.

As an important part of the now accepted role of mechanics in cell biology, forces applied specifically to the nucleus have emerged as key elements controlling cell function. Several force-driven nuclear sensing mechanisms have been shown in recent years and have also been summarized in recent reviews.^1–3^ To mention only a few recent examples, nuclear deformation alters chromatin rheology, thereby preventing DNA damage,^4^ and changes in nuclear shape trigger mechanosensing events that affect transcription.^5,6^ Among the different possible mechanical effects occurring at the nucleus, changes in nucleocytoplasmic transport, and specifically in the function of nuclear pore complexes (NPCs), are a particularly interesting possibility.

## EVIDENCE FOR A ROLE OF MECHANICS IN NUCLEOCYTOPLASMIC TRANSPORT

The potential role of NPCs in mechanotransduction was first discussed more than a decade ago,^7^ and the first evidence of forces affecting nucleocytoplasmic transport (by increasing it) was shown three decades ago.^8^ In addition, NPC permeability affects nuclear mechanical properties,^9^ and a few years ago, we described that force application to the nucleus is necessary and sufficient for the nuclear accumulation of the transcription regulator YAP.^10^ We rationalized this finding with the regulation of nucleocytoplasmic transport, since nuclear accumulation of YAP under force was inhibited when active transport through NPCs was impaired. Further supporting this, we also found that passive molecular diffusion through NPCs increased upon force application to the nucleus. Since then, other studies have confirmed the mechanosensitivity (i.e., force-dependency) of nucleocytoplasmic transport of different transcription factors,^3^ including YAP^11^ and MyoD.^12^ Importantly, force physically applied to the nucleus is not the only relevant mechanical parameter, as we also found that the resistance of nuclear-translocating proteins to deformation under force (i.e., their mechanical stability) affects their transport rate across NPCs.^10,13^ Despite this evidence, whether there is a force-related mechanism governing nucleocytoplasmic transport specifically (rather than retention of cargo molecules in the nuclear or cytoplasmic compartments), and specifically controlling both diffusive and facilitated transport, remains to be elucidated. Understanding the mechanosensitivity of nucleocytoplasmic transport may provide important principles by which forces control transcription through the localization of transcription factors, with clear implications in cancer biology, tissue engineering, and regeneration, among others.

## MECHANISMS OF NUCLEOCYTOPLASMIC TRANSPORT

**FIG. 1.**
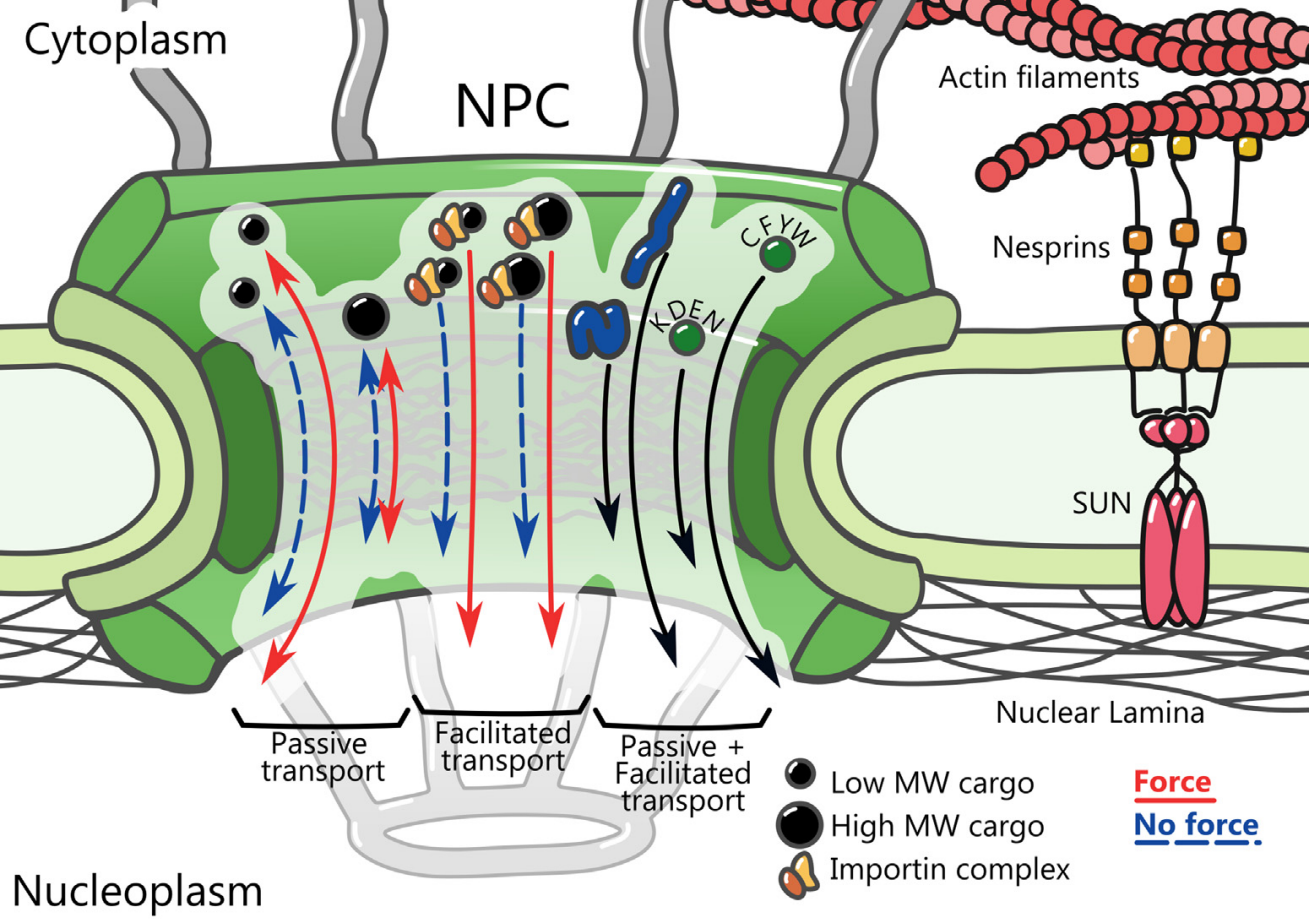
Mechanical effects in NPCs. NPCs are composed of cytoplasmic and nuclear filaments (forming the nuclear basket in the nuclear case, gray), the cytoplasmic and nuclear rings (light green), the inner ring that lines the central channel (dark green), and the FG-nup proteins that form the permeability barrier (gray). Force transmission to the nucleus can occur non-specifically (for instance, as cells migrate through small constrictions) or specifically through the LINC complex (formed by SUN and nesprin proteins, right), which links actin filaments to the nuclear lamina. Regarding the graphs in the central channel, from left to right: passive transport is increased by force applied to the nucleus, but only for small MW proteins. In contrast, active/facilitated transport (in which cargo proteins are bound to nuclear transport receptors such as importins) is affected regardless of MW. Diffusion through nuclear pores is also affected by protein mechanical stability, and the chemical composition of surface-exposed residues (marked with the different residues associated with low/high diffusion). In principle, the effect of these two factors should apply both for passive and facilitated transport. Although not tested, these two factors could also regulate mechanosensitivity in a manner similar to MW.

Nucleocytoplasmic transport is controlled by NPCs, which cross the nuclear membrane and control transport between the nucleus and the cytoplasm bi-directionally. NPCs are large macromolecular assemblies of different proteins (generally called nup proteins) with an eightfold symmetric distribution (Fig. 1).^14^ NPCs are composed of eight filaments at the cytoplasmic side, connected to a cytoplasmic ring complex, which is in turn connected to an inner ring. The inner ring is also connected to a nucleoplasmic ring complex at the nuclear side, which is linked to a structure termed the nuclear basket.^15^ The permeability of NPCs is controlled by proteins containing intrinsically disordered phenylalanine-glycine rich domains (FG-nups). FG-nups line the central channel of the NPC^16^ and form a selective barrier (called the permeability barrier) to nuclear/cytoplasmic exchange. Transport through NPCs occurs through two main modes, passive and facilitated transport.^14,17^ Passive transport refers to diffusion in and out of the nucleus, without any energetic cost. Due to the permeability barrier, passive diffusion rates gradually decrease with protein molecular weight (MW), meaning that proteins above 30–60 kDa have a very low probability of successfully diffusing from one end to the other of the NPC.^18–20^ This MW dependence can be explained by different mechanisms, such as the sieve-like properties of the NPC^20^ and its formation of an entropic barrier.^19^ Beyond MW (and more specifically, protein size), in recent years other molecular properties such as the chemical composition of surface-exposed residues^21^ and mechanical stability^10^ have emerged as important regulators of protein transport through NPCs (Fig. 1).

In contrast to passive transport, facilitated transport (also known as active transport) uses nuclear transport receptors to transport cargo,^22,23^ allowing for much faster traverse rates.^20^ In canonical facilitated import from the cytoplasm to the nucleus, proteins that show a nuclear localization sequence (NLS)^24^ can bind to importins α, which can then bind to Importin β. The latter acts as the master regulator of active import, by mediating fast passage through NPCs via specific interactions with FG-nups.^25^ A similar mechanism exists to traverse the NPC from the nucleus to the cytoplasm. Proteins undergoing facilitated export show a nuclear export sequence (NES) which binds to exportin proteins, which then form a complex with the GTPase Ran. This enables protein export, again via specific interactions between exportins and FG nups.^25^ The directionality of facilitated diffusion in the import or export direction is given by the hydrolysis of Ran. Ran GEF and GAP proteins localize, respectively, to the nucleus or cytoplasm, leading to a predominance of Ran GTP in the nucleus, and of Ran GDP in the cytoplasm.^23^ Nuclear Ran GTP mediates both the release of cargo from the import complex, and the formation and export of the export complex.^26^ In turn, cytosolic Ran GDP is able to bind to the importin NTF2, thereby translocating to the nucleus and closing the cycle. In the overall cycle, the actual crossing of NPCs does not require energy and occurs passively, and is in fact termed “facilitated diffusion.” The energy-consuming (and therefore active) step is the hydrolysis of Ran GTP, and the associated maintenance of a steep Ran GTP/GDP gradient on both sides of NPCs.^23^

## REGULATION OF NUCLEOCYTOPLASMIC TRANSPORT BY FORCE APPLICATION TO THE NUCLEUS

Increasing evidence now suggests that both passive and facilitated diffusion are regulated by force application to the nucleus. Regarding passive transport, we have previously used fluorescently labeled dextran of different MW to show that diffusion through the NPC is mechanosensitive:^10^ when the nucleus is under force, diffusion through the NPC is faster. A similar phenomenon was described by using GFP as a marker, and by comparing nuclei on cells seeded on flat substrates (where cells spread and flatten, leading to highly deformed nuclei likely submitted to high forces) vs three-dimensional scaffolds (where both cells and nuclei acquire more rounded shapes, likely experiencing lower forces). In this case, GFP diffused faster for cells on the flat substrates.^27^ Relatedly and also using GFP as a marker, recent work has reported decreased diffusion across NPCs for cells under cellular energy depletion conditions, a treatment likely to reduce cell contractility and thereby force application to the nucleus.^28^ Recently, using GFP-tagged artificial proteins of various MW, we have confirmed that diffusion through NPCs is faster under force^29^ (Fig. 1). Furthermore, the mechanosensitivity is higher for proteins with lower MW, and is progressively lost as MW increases. The higher mechanosensitivity of smaller proteins is consistent with a potential opening of the NPC (increase in diameter) caused by force. This opening would have a higher impact on proteins of smaller size than on bigger proteins, where the change in NPC size may not be sufficient to increase their low diffusion rates. Taken together, these findings show that forces to the nucleus decrease the permeability barrier of the nucleopore for passive diffusion.

Due to the involvement of the active, highly regulated nucleocytoplasmic transport cycle, the study of the mechanosensitivity of facilitated transport is more complex. Our previous work showed that the facilitated import of the mechanosensitive transcriptional regulator YAP is increased when force is applied to the nucleus.^10^ By comparing cells in two- vs three-dimensional substrates as explained above, it has been shown that mechanics control the nuclear accumulation of MyoD, by increasing its nuclear concentration.^12^ However, force-dependent transport of proteins can be due to different effects. First, force could induce changes in protein affinity for binding partners in the cytoplasm or nucleus, as described for other transcription factors such as MRTF-A,^30,31^ β-catenin,^32^ or twist-1.^33^ Second, force could affect signaling by regulating the coupling between cargo proteins and the nucleocytoplasmic transport machinery, for instance by exposing NLS/NES sequences and making them available for binding. Such a mechanism, mediated by actin and src-family kinases, has been proposed to regulate YAP export.^11,34^

Finally, transport itself could be constitutively affected by force, regardless of specific signaling events. To explore this hypothesis, we recently combined artificial proteins of various MW with NLS sequences with different affinities for importin α.^29^ These proteins did not have binding partners in either cytoplasm or nucleus or any regulation of their NLS sequence, and thereby directly probed the response of the nucleocytoplasmic transport cycle. These results showed that when forces reach the nucleus, facilitated diffusion through the NPC increased. Interestingly and unlike the case of passive transport, neither the magnitude nor the mechanosensitivity of facilitated diffusion depended on MW, at least until approximately 60 kDa (Fig. 1). This different behavior could be explained by the different cargo sizes that each type of diffusion is able to transport: whereas passive diffusion sharply decreases above 40 kDa, facilitated diffusion is able to transport very large cargos.^35^ Thus, it is likely that the loss of mechanosensitivity for facilitated diffusion appears at very high MW, where transport itself is affected.^35^ Regardless of its origin, the differential effect of MW on passive and facilitated diffusion means that for proteins of the right size that can undergo both passive and facilitated transport, mechanosensitivity is different for each type of transport. This enables a mechanosensitive nuclear accumulation of proteins, which depends both on MW and on the affinity of the NLS for importin α. Of note and although we have not yet tested it, mechanosensitive nuclear accumulation of proteins could likely be achieved by regulating passive diffusion not through MW, but through other factors mentioned above such as surface properties, or protein mechanical stability. Interestingly, this mechanism works not only for protein import (with NLS sequences) but also for protein export (with NES sequences), although the effect is milder in the export case. This may be related to volume differences of the nuclear and cytoplasmic compartments.

## TRANSMISSION OF FORCE TO NPCs AND ASSOCIATED CONFORMATIONAL CHANGES

Thus, forces applied to the cell nucleus constitutively affect both passive and facilitated diffusion, strongly suggesting some sort of force-induced conformational change in NPCs that affects the permeability barrier. The nature of this change, however, remains as an open question. Force exerted via actomyosin contractility reaches the nucleus, and specifically the nuclear lamina, through the linker of nucleoskeleton and cytoskeleton (LINC) complex^36^ (Fig. 1). From the LINC complex, forces could reach NPCs through the connections between the nuclear lamina and NUP153,^37^ or through the association of the LINC complex protein SUN1 to the NPC protein NUP153.^38^ Interestingly, mechanosensitive effects on transport are observed not only when forces reach the nucleus through the cytoskeleton and LINC complex, but also when forces are applied non-specifically by compressing nuclei with Atomic Force Microscope.^10,29^ This suggests that force-induced effects may not specifically require the LINC-NPC connection. This also means that transport may be regulated not only by contractile forces originating at the actomyosin cytoskeleton, but also by other types of forces (for instance, nuclear compression as cells migrate through constrictions).

Once force reaches NPCs, it may affect them in different ways. By using transmission electron microscopy and comparing cells plated on soft vs stiff substrates (where force transmission to the nucleus is increased), we found an increase in the apparent diameter of NPCs on stiff substrates.^10^ In a similar approach using scanning EM tomography, another study found differences in the maximum diameter of NPCs between rounded and highly spread cells, but not in total NPC area.^27^ This led authors to hypothesize that mechanically induced effects occur likely at the nuclear basket. Different publications analyzing NPC structure have confirmed that the NPC is a flexible structure that can change conformation, although dilation of the nucleopore is not needed for typical cargos to go through it.^39^ First, it was reported that the Y complex shows regions of great flexibility.^40^ Then a “ring-cycle” mechanism was proposed for the central transport channel of the NPC.^41^ This mechanism suggested that dilation and constriction can be achieved by rearranging the organization of the nup proteins assembling the channel from a thin, large ring to a thick, smaller cylinder. However, this rearrangement was observed in partial but not more complete reconstructions of the entire NPC molecular architecture, suggesting that it is not feasible in physiological conditions.^42,43^ Very recently, conclusive evidence on NPC deformability has emerged from cryo EM experiments. Indeed, NPCs were shown to increase in volume for NPCs on cells attached to a stiff substrate (as opposed to isolated NPCs,^44^ where force transmission to nuclei would be expected to be lower). Furthermore, perturbing nuclei by energy depletion and hyperosmotic shocks were shown to constrict the NPC central channel, by bringing together the spokes that line it.^28^ This effect is consistent with a mechanical increase in nuclear membrane tension, which would expand NPCs. Consistently with this hypothesis, changes in the diameter of the NPC central channel correlated with the separation between the inner and outer nuclear membranes (which should increase with membrane tension^28^).

## REGULATION OF TRANSPORT BY THE MECHANICAL PROPERTIES OF TRANSLOCATING CARGO MOLECULES

In addition to the physical, elastic deformation of the NPC upon force application, we recently uncovered an independent mechanism that endows the NPC with an extra layer of mechanosensitivity, based on the mechanical stability of the translocating proteins. Upon tagging a MRTF-A-GFP transcription factor with proteins with varying mechanical stabilities—independently measured using single molecule Atomic Force Microscopy—we found that the rate of nuclear import (and the amount of nuclear accumulation) was inversely proportional to the mechanical (and not the thermal) stability of the shuttling protein.^13^ In other words, mechanically labile proteins translocate to the nucleus across the NPC faster and more efficiently than mechanically stiff ones. Crucially, the mechanism seems to be universal and independent of MRTF-A. In fact, analogous experiments using a LEXY optogenetic tool^45^—hence independent of any transcription factor transporter—modified to incorporate proteins of varying mechanical stabilities led to the same conclusions, demonstrating that proteins with high mechanical stability exhibit impaired nuclear shuttling. From the fundamental perspective, these experiments suggested that, in addition to MW^19^ and to the chemical composition^21^ of exposed amino acids, the mechanical stability of proteins emerges as a new, complementary property that regulates the nuclear transport of proteins. From the applied viewpoint, one can envisage engineering the mechanical properties of transcription factors to regulate their nuclear accumulation, thereby affecting force-induced transcriptional programs and ultimately cell function. In fact, we showed that MRTF-A proteins modified to be mechanically stiffer resulted in a downregulation of SRF-related genes, with a subsequent significant decrease in the motility of U2OS, MDA-MB-231, and HeLA and cancer cells.^13^

## IMPLICATIONS AND PERSPECTIVE

Despite these exciting new findings, many questions on the role of protein mechanical stability remain elusive. Mechanistically, we still lack the answer to the central question of who applies the force to the protein to unfold in the proximity of the NPC; similarly, we do not know how mechanical force distributes across the backbone of complex multimodular shuttling proteins. In particular, we have no evidence of whether, similar to the (narrower) bacterial proteolytic ClpX^46,47^ machinery and mitochondrial^48^ pores, the (larger) NPC mouth senses the local mechanical stability of the translocating proteins, and whether the hierarchy^49,50^ in the mechanical stability of proteins probed in *in vitro* nanomechanical experiments is conserved in the complex NPC cellular context.

In summary, increasing evidence now shows that nucleocytoplasmic transport is constitutively affected by mechanical force, and regulated as well by the mechanical properties of the cargo molecules being transported. Several aspects remain to be addressed to understand this phenomenon, chief among them the related force-dependent structural changes in NPCs, the nature and origin of the forces applied to translocating proteins, and the interplay between both in principle orthogonal mechanisms. Understanding the force-induced effects in the NPC permeability barrier mediated by FG-nups will be particularly challenging, due to its highly dynamic structure. Besides this, different fascinating questions can now be posed: To what extent can this phenomenon explain the reported mechanosensitive nuclear localization of many proteins, and how is this coupled to other mechanisms? Have the design rules of protein mechanosensitivity (tuneable through properties such as MW, protein mechanical stability, or NLS/NES sequences) been harnessed by evolution to implement mechanosensitive signaling pathways? And equally excitingly, can we now use these rules to exogenously engineer mechanosensitivity in cell engineering applications? In our view, the emergence of the mechanosensitivity of nucleocytoplasmic transport, and of NPCs themselves, opens an entirely new perspective in mechanobiology. Until now, mechanotransduction pathways have typically been described as specific events affecting specific pathways. In contrast, if we achieve a systematic understanding of how mechanics affects transport, we will have a framework with which to rationalize mechanical effects across signaling pathways, and across physiological scenarios. We foresee that this will be the subject of intense research in the coming years.

## Data Availability

Data sharing is not applicable to this article as no new data were created or analyzed in this study.
